# Effect of Extraction Methods on Polyphenols, Flavonoids, Mineral Elements, and Biological Activities of Essential Oil and Extracts of *Mentha pulegium* L.

**DOI:** 10.3390/molecules27010011

**Published:** 2021-12-21

**Authors:** Mohammed Messaoudi, Abdelkrim Rebiai, Barbara Sawicka, Maria Atanassova, Hamza Ouakouak, Imane Larkem, Chukwuebuka Egbuna, Chinaza Godswill Awuchi, Sihem Boubekeur, Mohamed Amine Ferhat, Samir Begaa, Naima Benchikha

**Affiliations:** 1 Nuclear Research Centre of Birine, P.O. Box 180, Ain Oussera, Djelfa 17200, Algeria; samirbegaa@yahoo.fr; 2 Chemistry Department, University of Hamma Lakhdar El-Oued, B.P.789, El-Oued 39000, Algeria; rebiai-abdelkrim@univ-eloued.dz (A.R.); hamza39ouakouak@gmail.com (H.O.); Naima_chem@yahoo.fr (N.B.); 3 Department of Plant Production Technology and Commodities Science, University of Life Science in Lublin, Akademicka 15 Str., 20-950 Lublin, Poland; barbara.sawicka@up.lublin.pl; 4 Nutritional Scientific Consulting, Chemical Engineering, University of Chemical Technology and Metallurgy, 1734 Sofia, Bulgaria; 5 Agronomy Department, University of Mohamed Khider Biskra, P.O. Box 145, Biskrah 07000, Algeria; imene.larkem@yahoo.com; 6 Nutritional Biochemistry and Toxicology Unit, World Bank Africa Centre of Excellence, Centre for Public Health and Toxicological Research (ACE-PUTOR), Department of Biochemistry, University of Port-Harcourt, Rivers State 500001, Nigeria; egbunachukwuebuka@gmail.com; 7 School of Natural and Applied Sciences, Kampala International University, Kampala P.O. Box 20000, Uganda; awuchi.chinaza@kiu.ac.ug; 8 Research Development Centre, SAIDAL, 35 Benyoucef Khattab Avenue, P.O. Box 16000, Mohammadi, El-Harrah, Algiers 16000, Algeria; chimieanalytiquesi@gmail.com; 9 Higher Normal School, Kouba (ENS), P.O. Box 92, Algiers 16308, Algeria; ferhatamine100@yahoo.fr

**Keywords:** *Mentha pulegium*, essential oils, mineral, total phenol, biological activity, antimicrobial properties

## Abstract

Our study evaluated the in vitro antioxidant properties, antibacterial and antifungal activities, anti-inflammatory properties, and chemical composition of the essential oils (EOs), total phenol, and total flavonoid of wild *Mentha pulegium* L. This study also determined the mineral (nutritional and toxic) elements in the plant. The EOs were extracted using three techniques—hydro distillation (HD), steam distillation (SD), and microwave-assisted distillation (MAD)—and were analyzed using chromatography coupled with flame ionization (GC-FID) and gas chromatography attached with mass spectrometry detector (GC-MS). The antioxidant effects of the EOs were tested with 2,2-diphenyl-1-picrylhydrazyl (DPPH) and ABTS (2,2’-azino-bis(3-ethylbenzothiazoline-6-sulfonic acid), while the antibacterial and antifungal activities of the EO and methanolic extract were tested using *Staphylococcus aureus, Bacillus subtilis, Pseudomonas aeruginosa, Escherichia coli,* and *Candida albicans.* Twenty-six compounds were identified in the essential oil, representing 97.73% of the total oil, with 0.202% yield. The major components were pulegone (74.81%), menthone (13.01%) and piperitone (3.82%). Twenty-one elements, including macro- and micro-elements (Ba, Br, Ca, Cl, Co, Cr, Cs, Eu, Fe, K, Mg, Mn, Mo, Na, Rb, Sb, Sc, Sr, Th, U and Zn), were detected using neutron activation analysis (INAA) and inductively coupled plasma optical emission spectrometry (ICP-OES), with the concentration of mineral element close to the FAO recommendation. The results show that the EOs and extracts from *Mentha pulegium* L. had significant antimicrobial activities against the microorganisms, including five human pathogenic bacteria, one yeast (*Candida albicans*), and one phytopathogenic fungi. The in vivo anti-inflammatory activities of the leaf extracts were confirmed. The results indicate that the EOs and extracts from *Mentha pulegium* L. have promising applications in the pharmaceutical industries, clinical applications, and in medical research.

## 1. Introduction

Medicinal plants and their medicinal properties have been widely studied, especially in developing countries [1,2,3,4]. The World Health Organization has acknowledged the importance of medicinal plants, along with traditional medicine, which has received global attention [5,6]. In many countries, such as Algeria, there is a search for alternative medicine, including traditional herbal treatments. Many patients are not often satisfied with the treatments they receive using modern medicine, and as a result, prefer herbal remedies, which are natural, harmless, inexpensive, and have little or no side effects [7].

Recently, the demand for natural medicines for primary health care is increasing globally, causing manufacturers of herbal extracts and producers of essential oils to turn attention to medicinal plants and their phytoconstituents, using the most appropriate extraction techniques. Several different methods are used to produce extracts and essential oils of specific quality with active compounds from plant materials, even though the extraction of bioactive components from plants has always been a challenge for researchers. Among these widely used techniques for extracting the active compounds from the plant are steam distillation, hydrodistillation, microwave-assisted extraction, supercritical fluid extraction [8,9], ultrasound-assisted extraction, and counter-current extraction [10].

Pennyroyal mint, known as *Mentha pulegium L*., is a herbaceous flowering plant that belongs to the Lamiaceae family native to Europe and North Africa. The aerial parts of *M. pulegium* have been used as herbs since ancient times for treating diseases, including digestive disorder, as a snack, and as infusion to prepare a coffee-like drink [11,12]. The phytochemical composition of *M. pulegium*. has been examined in some studies, with less attention given to comparing the chemical composition, including the mineral content, of its essential oil and the whole plant using a different method of extraction. In addition, the valuable secondary metabolites’ in vivo inflammatory activity has not received much attention. A few studies have described total flavonoid, phenol content, and antioxidant activity of the aerial parts of *M. pulegium* from the semi-arid region of Algeria.

There are many studies focusing on the biological and pharmacological studies of plants (aerial and underground parts) in folk medicine [13,14,15]. Several studies on medicinal plants have reported that there are marked differences in chemical composition (total flavonoid, total phenolic contents), mineral count, and biological activity due to differences in geographical regions and locations across the world. However, there is increasing need to provide a scientific basis for the traditional use of different plants and plant parts. For this reason, this study aimed to provide in-depth information on the extraction of essential oil, methanolic extract, biological activities, and mineral compositions of *Mentha pulegium* L. It is part of an overall program in our laboratory for studying natural food samples relevant to human health and nutrition [16,17,18,19,20,21]. The results of this study will be useful in enriching the database of Algerian medicinal herbs, advancing the knowledge on medicinal plants, and deepening the scientific understanding of the medicinal, elemental, and bioactive compounds and antimicrobial properties of *Mentha pulegium*.

## 2. Material and Methods

### 2.1. Experimental Analyses

#### 2.1.1. Collection and Preparation of Plant Sample

The aerial parts of *Mentha pulegium* L. were collected in June 2018 from El-Guetfa region, Msila, located in the semi-arid region of Algeria (35°44′26″ N, 3°3′05″ E). Firstly, the samples were washed several times with deionized water and were dried for two weeks at room temperature. The dried samples were ground to a fine powder (particle size fraction of <200 μm) using an agate mortar and pestle.

#### 2.1.2. Mineral Element and Calculations

In this work, for determination of the mineral element concentration, the techniques of INAA (Instrumental Neutron Activation Analysis) and ICP-OES were used. Firstly, for the INAA technique, three samples of each collected charge weighing about 200 mg were stored in precleaned polyethylene capped bottles. We used the certified reference materials as NIST-SRM from the National Institute of Standard and Technology (NIST). All the samples and standard were packed and irradiated together for 6 h at a thermal neutron flux of 4.5 × 10^13^ cm^−2^s^−1^. After appropriate cooling, the irradiated samples together with the standard were measured at different cooling times using a coaxial HPGe detector having the following characteristics: relative efficiency of 35% and FWHM 1.8 keV for the 1332.5 keV γ-peak of ^60^Co. At the end, we determined the concentrations of the elements (major and trace elements) using the equation of INAA given by the Equation (1) [17].

(1) $$\rho \left(\text{µ}g/g\right)=\frac{\;{\left[\left(N_{p}/t_{c}\right)/DC\right]}_{a}}{{\left[\left(N_{p}/t_{c}\right)/DC\right]}_{s}}\frac{{\left(\rho W\right)}_{s}\;}{W_{a}}$$

where the subscripts *a* and *s* refer to the sample and the standard, respectively, *N_p_* is the net photo-peak counts, *W* is the sample mass, *D* = [exp (−λ t_d_)] is the decay factor, and *C* = ([1 − exp (−λ t_m_)]/λ t_m_) is the counting factor, *λ* decay constant, t_c_, t_d_ and t_m_ counting, decay and measurement times, respectively.

To check the accuracy of the analytical method, we determined the parameter of U-score test, calculated according to the Equation (2):

(2) $$U_{\mathrm{score}}=\frac{\left|X_{\mathrm{Lab}}-X_{\mathrm{Ref}}\right|}{\sqrt{\mu_{\mathrm{Lab}}^{2}+\sigma_{\mathrm{Ref}}^{2}}}$$

where X_Lab_, μ_Lab_, X_Ref_ and σ_Ref_ are the laboratory results, standard deviation, the recommended, and standards uncertainties, respectively.

The laboratory performance is evaluated as: satisfactory if U score ≤ 1, and unsatisfactory for U score > 1 (result and certified value are not in agreement) [22].

Secondly, by using the technique of ICP-OES, three samples of each collected charge weighing about 500 mg were added and digested following the protocol of our laboratory [20,23], after the full dissolution of our simple, on letting the solution homogenize by settling. Then, the samples were analyzed using ICP to determine the mineral content.

#### 2.1.3. Extraction of the Essential Oil and the Analysis

*Mentha pulegium* essential oils (EO) were extracted in three ways—hydrodistillation, steam distillation and microwave distillation—for the aerial part of the plants studied. The EOs obtained were then analyzed using the GC-FID and GC-MS techniques. In order to determine its chromatographic profile, the different compounds were listed according to their retention times (Tr). The samples studied in this work were subjected to distillation either with water to prepare hydro-distillation essential oil (HD-EOs), or steam to prepare steam distillation essential oil (SD-EOs) for three hours using the Clevenger-type apparatus. The simples (100 g each) were subjected to the third method of distillation with microwave-assisted distillation, using a flask with 250 mL of distilled water, and placed in home appliance microwave (Model: Midea AG823ABI) with few modifications to accommodate the Clevenger-type device. The time was set at 30 min and operated at 800 W, as adapted earlier [24]. All the EOs were collected and dehydrated over anhydrous sodium sulfate (Na_2_SO_4_) to eliminate water. After that, they were stored at 4 °C in a sealed structure of glass vials until analysis. The analysis and determination of the chemical molecules of essential oil were done using gas chromatography–flame ionization (GC-FID) equipped by column Rtx-5MS fused bonded column with properties (5% phenylmethylpolysiloxane, 30 m × 0.25 mm i.d., 0.25 µm film thickness). The carrier gas was nitrogen at a rate of 1 mL/min and programming temperature of 60 °C for 5 min, then increasing at a rate 3 °C/min to 250 °C, after which the detector was adjusted to 300 °C. In this experiment, the volume of injection was 0.1 µL. The ratio of each individual component was obtained from electronic integration. Moreover, using the mass spectral analysis, we analyzed the EOs using gas chromatography–mass spectrometry (GC/MS), which were characterized by the sign of Shimadzu GCMS-QP2010 (Tokyo, Japan) equipped with Rtx-5MS fused bonded column (30 m × 0.25 mm i.d. × 0.25 µm film thickness) (Reset, 2124 Fillmore St., San Francisco, CA, USA) directly coupled to a quadrupole mass spectrometer, with an initial temperature of 45 °C for 2 min, followed by increasing at a rate of 5 °C/min to 300 °C and then kept for 5 min. The carrier gas was helium at rate of 1.5 mL/min, while the mass spectra condition was FEC = 60 mA (filament emission current), IS = 200 °C (Ion source), IV = 70 eV (Ionization voltage) and diluted samples were 1% *v*/*v*. For identifying the essential oil chemical components, we made a comparison between their experimental spectra, with the spectral mass spectroscopy in the MS Library (NIST and WILEY), in parallel calculation of the retention index (RI) compared by RI for Adams 2007.

#### 2.1.4. Preparation of the Methanol Extract

The extraction was done according to the previous method [19]. The leaves of *Mentha pulegium* L. were extracted three times with methanol–water (80:20, *v*/*v*). After concentration under reduced pressure using a rotary evaporator (Rotary Evaporators type Büchi Rotavapor® R-II, R-210, R-215), the extracts were stored at 4 °C for further use.

#### 2.1.5. Total Content of Phenol and Flavonoids

The determination of the total phenolic content was done spectrophotometrically using the Singleton–Rossi method with the Foolin–Ciocalteu reagent as described by Müller et al. [25]. The results were expressed as milligrams of gallic acid equivalents per gram of extract (mg GAE/g) from a calibration curve with gallic acid. The sample was measured in three replicates.

The flavonoids were done using the spectrophotometric method of the Singleton–Rossi method with the Folin–Ciocalteu reagent. The wavelength λ was 765 nm, and by help of gallic acid graph as reference, we determined the total phenol content. The total flavonoid content was determined using the method reported by Topçu et al. [26], and the results were reported in micrograms of quercetin equivalent per gram of extract (mg QE/g). The sample was measured in three replicates.

### 2.2. Biological Evaluation

#### 2.2.1. Antioxidant Activity Using DPPH Radical-Scavenging Activity

The DPPH free radical-scavenging activity of the extract and essential oil samples were estimated according to the previously reported method [27], with slight modification using the stable DPPH radical, which has an absorption maximum at 515 nm. It was experimentally done by taking 0.5 mL from each sample (oil or extract) and mixing it with 1 mL of DPPH (0.2 mM), after which they were left in the dark at room temperature for approximately 30 min, and then measured at the absorbance of 517 nm. The inhibition ratio was calculated as follows:

I% _(DPPH)_ = ((A_0_ − A_1_)/A_0_) × 100 (3)

where A_0_ was the absorbance of DPPH solution and A_1_ was the absorbance of sample. The results were represented as IC_50_ values.

#### 2.2.2. ABTS Radical-Scavenging Activity

The ABTS free radical scavenging activity of essential oil sample was determined by ABTS radical cation decolorization assay [27]. The experiment was conducted following the method described in the DPPH Scavenging Activity Section. Measurements were then gradually carried out in triplicates. The percentage of inhibition of absorbance at 734 nm was calculated using the following formula:

I% _(ABTS)_ = ((A_0_ − A_1_)/A_0_) × 100 (4)

where A_0_ was the absorbance of ABTS solution and A_1_ was the absorbance of sample. The results were represented as IC_50_ values

#### 2.2.3. Antibacterial and Antifungal Activities

To evaluate the antibacterial activities of the essential oil and the methanol extract of the plant, we used the paper disc diffusion method, including two Gram-positive strains (*Staphylococcus aureus ATCC 6538* and *Bacillus subtilis ATCC 6633*) and two Gram-negative strains (*Pseudomonas aeruginosa ATCC 9027* and *Escherichia coli ATCC 8739*), as well as one fungal strain (*Candida albicans ATCC 10231*). The tested strains were provided by RDC-SAIDAL, El-Harrah, Algeria. We prepared a paper disk of 6 mm diameter impregnated with 35 µg of essential oil or methanol extract solution and manually applied it on the surface of the agar plates inoculated with the microorganisms. We used reference standards such as fosfomycin, carbenicillin, erythromycin, and cephalexin with 35 µg/disk to determine the sensitivity of Gram-positive bacteria species; however, we used one reference standard, fosfomycin, for Gram-negative bacteria species. Before measurement, we left plates with paper disk on room temperature for 2 h at 37 °C to allow diffusion.

#### 2.2.4. In Vivo Anti-inflammatory Activity

**Figure 1:**
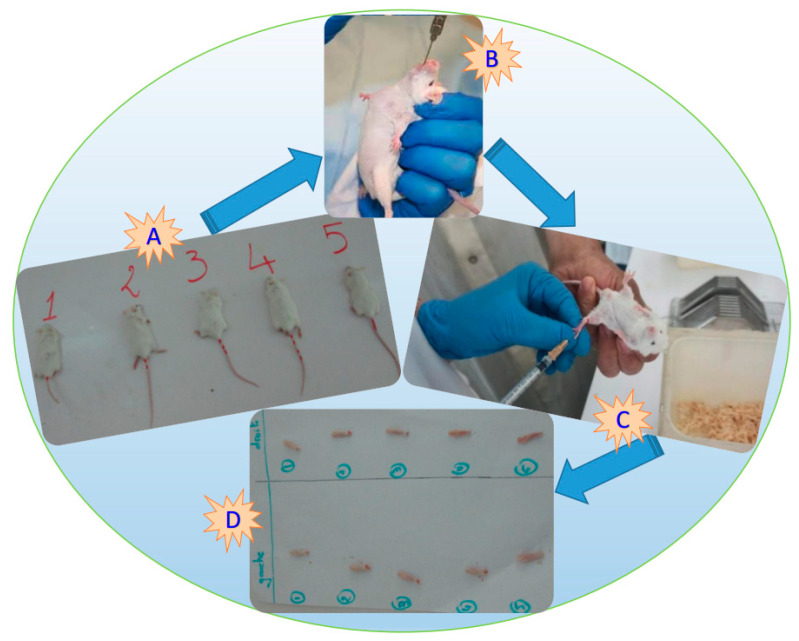
Photograph showing an albino rat for plant extract testing (**A**,**B**), the sub-plantar injection of carrageenan into the left posterior paw of a mouse (**C**), and the left and right hind legs cut at joint height (**D**) (all, original photos).

The various in vivo anti-inflammatory activities were done using albino mice of Swiss strain from the Pasteur Institute (Algiers). Male mice weighing between 20 and 22 g were used, and they were housed in plastic cages at room temperature (25 °C), lighted for 12 h per day. During the acclimatization period (a week before being used in the various experiments), the mice had free access to water and food (croquettes from the Animal Feed Production Company, Bouzareah, Alger). The in vivo anti-inflammatory activities were assessed by the method of inhibition of mouse paw edema induced by carrageenan. the principle of this study was injecting carrageenin under the plantar fascia of the left paw of the mouse to cause an inflammatory reaction that can be reduced by an anti-inflammatory product (Figure 1). This study allowed the comparison of plantar edema after administration of equal doses of the anti-inflammatory product to be tested (the extract of the plant at 10%) and the corresponding reference product (diclofenac sodium at 10 mg kg^−1^).

According to the experimental protocol Colot M. 1972 [28], we made the following step:

First step: At time T_0_

The mice were divided into 3 batches, each batch containing 5 mice randomly selected (see Figure 1A,B):

The control group: each mouse received 0.5 mL of physiological water.

The control group: each mouse received a reference anti-inflammatory drug, diclofenac at 10 mg kg^−1^.

The treated group received the test solution *Mentha pulegium* L. (MP). Each mouse received 0.5 mL of the plant extract at 10%.

One MP test batch: (5.0008 g 50 mL^−1^ H_2_O) × 100 = 10.00%.

Before starting the experiments, the mice were fasted for 16 h and after that weighed, then we administered intragastrical (gavage) for the five batches following suspensions (control solution, reference, and aqueous extract of the plant).

Second step: time T_0_ + 30 min:

Half an hour after dosing, mice from the five batches received 0.025 mL of 1% carrageenan under the plantar fascia of the left hind paw of the mouse (Figure 1C).

Third step: time T_0_ + 4H:

Four hours after this operation, the animals were sacrificed by breaking the neck, then the hind legs were cut at the height of the joint (Figure 1D), then weighed on an analytical balance.

The anti-inflammatory activity was calculated as a percentage reduction in edema in the treated mice compared to the control according to the following formula:

% inhibition of edema = ((PG − PD) _control mouse_ − (PG − PD) _treated mouse_)/((PG − PD)_control mouse_) (5)

where: PD = right leg weight, and PG = left leg weight

### 2.3. Statistical Calculations

The obtained results were statistically analyzed using one- and two-way ANOVA model (SAS 9.2 2008 model) [29] and multiple T-Tukey tests, with the adopted significance level α = 0.05. An analysis of variance model with main effects and interactions of the studied factors was used, and the detailed analysis focused mainly on the main effects and two-way interactions. T-Tukey’s multiple comparison tests enable detailed comparative analyses of means by distinguishing statistically homogeneous groups of the mean (homogeneous groups), determined with the use of the least significant differences (LSDs). The tables of the results contain the most critical elements of the analysis of variance by providing the calculated probabilities (the *p*-value) related to the F-test functions used (F-Snedecor or Fisher-Snedecor). The calculated *p* values determine the significance and size of the influence of the examined factors on the differentiation of the results of the analyzed variables by comparing them with the most commonly accepted significance levels α (0.05). The letter indicators at the mean (significance groups) define the homogeneous, statistically homogeneous groups. The presence of the same letter index with the mean (at least one) means no statistically significant difference. Successive letter indices a, b, etc., define groups of means in ascending order. Descriptive statistics were used as the first and basic step in analyzing the collected data in STATISTICA. The purpose of using descriptive statistics methods was to summarize the dataset and to draw basic conclusions and generalizations about the dataset.

## 3. Results

### 3.1. Mineral Elemental Analysis

The mean values of mineral elemental concentrations evaluated in *Mentha pulegium* L. are shown in Table 1. (±) represent the standard deviation calculated on the basis of triplicate analyses, multiple irradiations, or different photo peaks.

The elements with the greatest stability were Ca, Na, K, Cl, and Fe, while the elements with the greatest variability in the mineral composition of this species were Sc, Cs, E, Co, U, Sb, and Mo. The median, called the median value of the set, divides all our observations into two equal numbers of observations in the group: scores lower than the median and scores higher than the median. So, the median value tells us that half of our results were below the median value and the other half were above the median value. Range is the simplest measure of the scattering of results (variability). It is the difference between the maximum and minimum value in our set of observations. It only shows us what is the scope of our observations, but it does not inform what is happening “in the middle” of this range, e.g., what value was most frequent, or what is the average for our set of observations (Table 2). Standard deviation measures the variability of the results observed and tells us how much the results “change”, i.e., whether the scatter of the results around the mean is small or large. The highest values of standard deviation from the mean were noted for potassium, sodium, and iron, while the lowest values were for Sc, Sb, U, Co, Cs, Eu, Mo, and Th. Skewness is a measure of the symmetry or asymmetry of a distribution. If the distribution is perfectly symmetrical, the skewness value is zero. On the other hand, its negative values indicate a left-skewed distribution (the left leg of the distribution is elongated), and positive values—a right-skewed distribution (the right leg of the distribution is extended). Elements such as Br, Ca, Cs, Fe, Mg, Mo, Sc, and U were characterized by a left-skewed distribution, while the rest—right-skewed, with an elongated right arm of the distribution (Table 2).

### 3.2. Chemical Composition of Essential Oil M. pulegium

A total of 26 compounds were identified in *Mentha pulegium* essential oils. Pulegone was the main, most important component of the EO (74.81%), followed by menthone (13.01%), piperitone (3.82%), limonene (1.55%) and cis-isopulegone (1.31%). The other molecules are present but at a rate of less than 1% (Table 3). The highest yield of essential oils (yield of essential oil [*v*/*w*]% = 0.204) and the best composition of essential oils (total identified—97.73%) were obtained by the hydrodistillation method. The lowest values of these characteristics were obtained by microwave distillation (MAD).

The essential oil obtained in the hydrodistillation process was characterized by a light yellow color, its density at 35 °C was 933.9 mg ml^−1^, and the refractive index was 1.476.

From Table 4, it can be noted that there was a slight difference in the oil yield; it was almost equal in HD and SD (0.204% and 0.2%, respectively) and significantly less than that obtained using the MAD technique (0.175%). In the composition of mint oils, the largest share was pulegone (on average 73.17%), and the smallest—menthol (0.11%). The oil distillation technique had a significant impact on the composition of essential oils, with the exception of menthol. The highest contents of limonene, menthone and pulegone were obtained from the distillation carried out with the HD technique, and the highest amount of piperitone was obtained with the SD technique. The MAD technique turned out to be the least effective technique for obtaining essential oils (Table 4).

In addition, it was noted that the predominant components were the oxygen monoterpenes (93.99%, 89.38%, 77.98% for HD, then SD, then MAD, respectively), followed by hydrocarbon monoterpenes (from 0.36% to 2.54%), while for sexoterpenes, they were in very low percentages (less than 1%).

Table 4 compares our result to those obtained by other authors using different methods

### 3.3. Total Phenol and Total Flavonoid Content of M. pulegium Extract

Table 5 shows the total content of phenols and flavonoids obtained in this experiment. Firstly, it can be notable that, from this data, the plant contains considerable quantities of phenolic compounds and flavonoids. In this work, a non-exhausting cold maceration extraction method was used, and we obtained sufficient yields to achieve our study objective, and the rate of methanolic extraction for the aerial parts of *Mentha pulegium* L. was obtained at about 15.17%.

Descriptive statistics of phenolic compounds, flavonoids and their extraction rates indicate the high stability of these features (V = 3.55–6.06%, respectively) (Table 6). The median divides all our results into two equal group, i.e., results lower than the median and results higher than the median. In other words, the median value tells us that half of our results were below the median value, and the other half were above the median value. The standard deviation for phenolic compounds was 1.48, and the SD for flavonoids was 0.72. Skewness measured the asymmetry of the results obtained. It tells us how the results for a given variable were around the mean. Whether most of the observed results are on the left side of the mean, near the mean value, or on the right side of the mean. Skewness for all features turned out to be left-handed. The range is the difference between the maximum and minimum value from our set of observations and tells us what the range of our observations was. The difference between the maximum and minimum value was small, which proves the stability of these features. The coefficient of variability of the examined features, which is a relative measure of variability, independent of their titer, allows for an unbiased assessment. The low value of the coefficient of variation of phenolic compounds and flavonoids in the *Mentha pulegium* extract proves a very low variability of the analyzed traits and the homogeneity of the studied population.

### 3.4. The Biological Assessment 

#### 3.4.1. Antioxidant Activity Determined by DPPH and ABTS Radical Scavenging Activity Test

**Figure 2:**
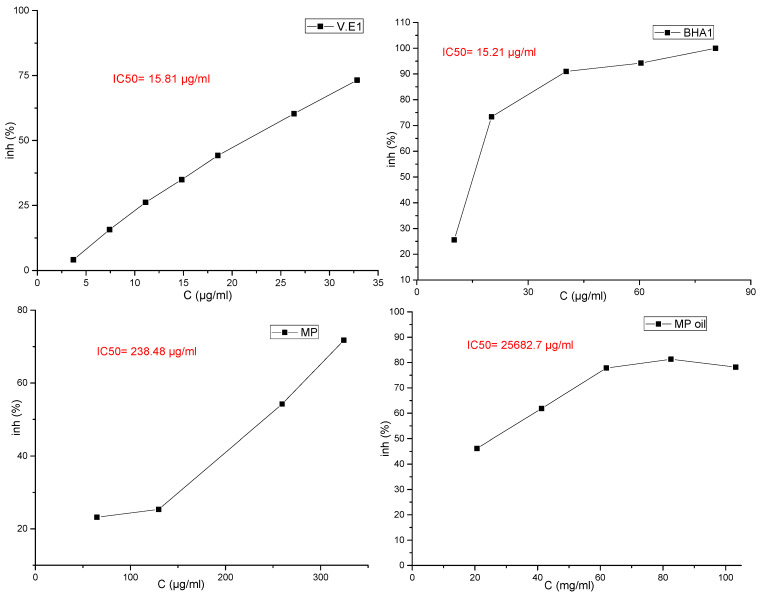
Curve of the antioxidant capacity of essential oils and crude extracts of the plant studied by the DPPH method with reference standards.

**Figure 3:**
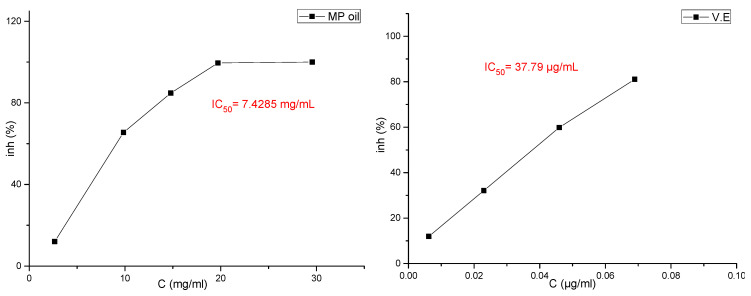
Curve of the antioxidant capacity of essential oils of the plant studied by the ABTS method with reference standards.

The results for the evaluation of functional properties expressed as free radical (2,1-diphenyl-2-picrylhydrazyl free radical (DPPH∙) activity and ABTS radical-scavenging activity are presented in Figure 2 and Figure 3 and in Table 7, respectively.

Several studies have shown the possibility of using essential oils as inexpensive sources of natural antioxidants in food products, after evaluating the degree of toxicity of these oils, in order to replace artificial antioxidants that have undesirable health effects [27]. El-Ghorab et al. [30] mentioned that it is not easy to identify the compounds responsible for the effects of radical scavenging due to the presence of many of them in the same essential oil, and many studies have indicated that the synergy of the oil compounds with each other makes the oil have a different and stronger effect than the effect of the dominant compounds when tested [31,32]. In the *M. pulegium* methanol extract, the high antioxidant activity may be explained by the high content of flavonoids (77% of total phenolics), whilst in essential oil the antioxidant activity may be explained by the high content of oxygenated compounds (93.99% of total chemical composition). This difference is understandable since all of these compounds are often reported [33] as having important antioxidant activities. In this study, it was shown that the results of the effectiveness of the ABTS test were better than the results of the DPPH news (IC_50_ = 7428.5 and IC_50_ = 25,682.7, respectively), and the results of the DPPH test and the ABTS test showed that the high IC_50_ value had a weak antioxidant activity for the essential oil compared to the reference compounds (Table 7).

In fact, based on the values of IC_50_ (Figure 2), the antioxidant activity in *M. pulegium* was lower compared to previous findings (17.92–25.66 µg/mL) [34,35]. The difference between our results and the others reported in literature may be attributed to the differences in chemical compositions, which depend on the areas of the plant collection, plant parts used and the extraction method, as acknowledged in a recent publication [36]. Despite these differences, the antioxidant activity of the *M. pulegium* oil and extract remains higher than activities of *Mentha rotundifolia* and *Mentha villosa*. The high antioxidant activity found for both extract and essential oil are critical and determinant for their therapeutic value and this may be in part responsible for their reputation as anti-inflammatory, hypotensive, hypoglycemic, and depurative agents.

#### 3.4.2. Antimicrobial Susceptibility Assay In Vitro

Antibacterial activity tests were performed on four Gram-positive and Gram-negative bacterial strains. The extracts prepared were from three examples. The results of the evaluation of the antibacterial potential of the extracts are shown in Table 8.

We evaluated the antibacterial activity of EOs and or methanolic extracts by the solid medium diffusion method using Mueller–Hinton medium for bacteria and Sabered medium for yeasts. The antibacterial activity was determined in terms of the diameter of the zone of inhibition (in mm) produced around the discs after incubation under conditions suitable for the development of the test germ.

This activity was revealed on four bacterial reference strains (*Staphylococcus aureus*, *Bacillus subtilis*, *Pseudomonas aeruginosa* and *Escherichia coli*) and a fungal strain (*Candida albicans*)

In the experiment, the inhibition zone showed that the activities of the essential oils HD and SD were very strong (very high sensitivity to bacteria) compared with the extract in both the bacterial Gram-positive and Gram-negative strains. However, in yeast, it was the opposite, the effectiveness of the extract was better than the oils, but when comparing the results with the reference compounds (fosfomycin, carbenicillin, erythromycin, cephalexin), we noted that the effectiveness of the oils is almost equal to the results of the reference.

For *Staphylococcus aureus,* the EOs of HD and SD were 37.33 and 24.33 mm, respectively, compared with the reference compounds (fosfomycin and carbenicillin), 44 and 37.5 mm, respectively. Moreover, for *Bacillus subti*lis, they were 25.5 and 26.3 mm for HD and SD, respectively, with 32.5 and 31 mm for the reference compounds (erythromycin and cephalexin), respectively. For the Gram-negative bacteria, the results were good in *Escherichia coli* at 31.7 mm for EO (HD) and 25.7 mm for EO(SD) compared with the reference compound fosfomycin at 33 mm.

The results of the activity of EOs HD and SD *Pseudomonas aeruginosa* bacteria were lower, at 16.7 and 13.7 mm for HD and SD, respectively, and the reference compound (fosfomycin) was 31 mm.

With yeasts, the results were relatively weak. We obtained 11 and 9 mm inhibition areas for HD and SD oil, respectively. For the crude extract of the plant, the results of inhibition were close to all types of microbes (bacteria and yeast) and ranged between 10.17 and 16 mm, which are results indicating the medium effectiveness of the extract compared to the essential oils.

After conducting the inhibition zones experiment, we noticed that the results were good in two types of Gram-positive bacteria (*Bacillus subtilis* and *Staphylococcus aureus)* and one type of Gram-negative bacteria (*Escherichia coli*). We decided to continue studying the minimum inhibitory concentration (MIC) and the minimum bacterial concentration (MBC) on these species. From bacteria, Table 8 shows the results obtained, where we noted that the best activity of SD oil was in the third bacteria *Staphylococcus aureus* (MBC = 5 and CMI = 2.5), while the HD oil had the best activity in the second bacteria *Escherichia coli* (MBC = 5 and CMI = 5). In the rest of the bacteria, the results of (MBC and CMI) were between 1 and 2 µL/mL.

When comparing these results in Table 8, with the results of other research [37,38,39], for the same type of plant, we noticed that the results obtained were similar to the ones obtained in our study for these bacterial strains. The oil is considered to be very effective against bacteria in general.

#### 3.4.3. In Vivo Anti-Inflammatory Activity

Table 9 groups all the results of the anti-inflammatory activity obtained

Oral administration of the aqueous extract of aerial parts of *Mentha pulegium* L. to mice with carrageenan-induced hind paw oedema produced significant (*p* < 0.001) anti-inflammatory activity at doses tested. Our study shows that the aqueous extract of *Mentha pulegium* L. significantly reduced carrageenan-induced paw oedema, when compared to the reference drug, diclofenac sodium (10 mg/kg). There was a gradual decrease in oedema paw weight to 22.16%, and the drug diclofenac sodium exhibited 52.97% inhibition. The result indicates that the effect of the extract is significant. These pharmacological properties exhibited by *Mentha pulegium* may be due to the presence of phenolic compounds.

## 4. Discussion

The data presented in this experiment show that nine essential micronutrient elements were present in *Mentha pulegium*, at K > Na > Fe > Mg > Mn > Zn > Cr > Mo > Co. Three potentially toxic elements were present (Br, Sb, and Th). In addition, nine other non-essential and non-toxic elements were identified. The mineral elements are very important, especially the micro- and macro-nutrients, which are essential to various human metabolic processes, and significantly contribute to human health [40].

The essential elements (Na, Fe and K) [41] were detected as the highest level among other elements. Moreover, K was determined and had the highest level (14,216 mg/kg), followed by Na (10,790 mg kg^−1^) and Fe (1604 mg kg^−1^). The result of the essential elements, such as zinc, chromium and cobalt are promising [41], with their concentration ranging from 0.77 to 44.5 mg/kg^−1^. Although all the three elements were present below the limits compared with the recommended values (RDA) [42].

Flavonoids are the most abundant chemical group in medicinal plants as secondary metabolites. The total flavonoid in methanolic extract of *Mentha pulegium* L. (Table 10) was recorded spectrophotometrically as 18.77 mg QE/g extract. The content of flavonoids compared to total polyphenols in *M. pulegium* was about 77%, whereas the other previous studies reported contents of flavonoids exceeding 90.45% of the total polyphenols [43].

Most studies have indicated that menotropin oxygen is dominant in the essential oil components of *M. pulegum* (Table 11), and that the predominant compounds are pulegone, menthone and piperitone. This is in line with our findings, as shown in Table 3. However, the proportions of these compounds differ from one region to another and have also been reported by these studies to be due to the difference in climate (rainy-moderate-dry), soils, and altitude above sea level [49,53], as well as the harvest period (before-after), flowering, and the part of the plant extracted from (twigs-leaves-flowers), as well as the extraction methods, HD, SD, MAD HD-CO_2_ [27,54,55], and age of the plant (first-second-third harvest).

The amount of extracted phenolics depends on some parameters, including the extraction temperature, time, pH, and solvent polarity [57,58]. The results of other authors may vary moderately from ours due to these factors. Additionally, studies have reported that the aerial parts of plants used for the extraction are of great importance because of different patterns of secondary metabolites [59]. For this purpose, water and methanol are considered the effective solvents for the extraction of the polyphenol constituents from plants [36,60]. The methanolic extract had high phenolic content of 24.49 mg GAE/g extract. A high total phenolic content in any extract indicates its high antioxidant capability. Comparing the previous works of literature (Table 11), Politeo et al. reported a total phenol content of 157.92 mg mg/g gallic acid equivalents (GAE)/g extract of *Mentha pulegium* L. in methanolic extracts [15]. It is more than the phenolic content in this study. Moreover, larger quantities were reported by Hajlaoui et al. and Sarikurkcu et al., 37.4 and 97.2 mg/g gallic acid equivalents (GAE)/g extract, respectively [43,56]. Benabdallah et al. found a TPC 17.00 mg/g gallic acid equivalents (GAE)/g extract [35]. The values of phenolic content in this current study varied compared to those in the literature. This may be due to the geographical variation or different methods of extraction, which may alter the amount of phenolics.

In a survey of past literature reports on the content of flavonoids (Table 11), it was found that Hajlaoui et al. reported a TFC of 33.83 mg QE/g extract of *Mentha pulegium* L. [43]. Politeo et al. determined a TFC of 18.58 mg QE/g extract of Mentha pulegium L. [11], and Sarikurkcu et al. found the TFC of 20.88 mg QE/g extrait [35,56]. The values of flavonoid content in this study varied slightly compared to those in the literature. As reported in the literature, biological and genetic diversity, and seasonal, environmental and year-to-year variations significantly impacted the flavonoid content of plants.

The measures of variability (measures of dispersion or dispersion) are necessary in research to measure the level of variation in the value of features in the studied community. It can be expressed in natural or ratio units. The statistics distinguish two groups of variability measures [6,36]—absolute measures of variability (nominated) and relative measures of variability—and they depend on the average value of the studied feature. The relative measures of variability include the coefficient of variation. The coefficient of variation allows one to assess the strength of differentiation of a given statistical community, demonstrating the strength of the variable, as well as to assess the arithmetic mean. A high value of the coefficient indicates a strong differentiation, and vice versa. In the conducted research, a very low coefficient of variation was obtained for the examined features. This means a very high stability of the yield of essential oils, analyzed mineral compounds, as well as the chemical composition of essential oils obtained by various determination techniques.

## 5. Conclusions

This study reports the phytochemical analysis, mineral elements, biological potentials, and effect of extraction methods on the chemical compositions of essential oil of aerial parts of the plant *M. pulegium* L., a wild-growing plant in the Algerian high plains. The essential oils were extracted by three methods, hydrodistillation, steam distillation and microwave distillation. The HD and SD were better in extraction yield and quantification of the major compounds, but the MAD was better in terms of extraction time, 30 rather than 180 min.

The mineral elements are very important, especially the micro- and macro-nutrients, which are essential to various human metabolic processes, and significantly contribute to human health. In this work, the mineral analysis of the plant studied through two techniques showed that *M. pulegium* is rich in minerals necessary for humans, such as calcium (31,875 mg/kg), potassium (14,216 mg/kg), iron (1604 mg/kg), sodium (10,790 mg/kg), magnesium (71 mg/kg), manganese (71.2 mg/kg), Zn (44.5 mg/kg), etc. In addition, the contents of the potentially toxic elements were well below the toxicological reference values compared to the tolerance limits set by the World Health Organization, giving this plant its special pharmaceutical and medicinal properties.

Based on the result obtained, it was shown that the antioxidant activity of the *M. pulegium* methanol extract is higher than that of the essential oil. However, it is a little low compared to the positive controls (BHT) and V.E. In vitro antimicrobial sensitivity assay showed that the activity of the essential oils, HD and SD, has a very high sensitivity against bacteria compared with the reference compounds (fosfomycin, carbenicillin, erythromycin, cephalexin), but the methanol extract had lower effects. The results of in vivo anti-inflammatory activity show that the methanolic extract of *M. pulegium* has a considerable and significant anti-inflammatory activity compared to the reference diclofenac sodium. The flavonoids and phenols may be responsible for the anti-inflammatory activity. These results indicate that *M. pulegium* methanolic extracts and essential oil may be useful as biological active agents in food and pharmaceutical formulations, because of their rich content of essential minerals.

## Figures and Tables

**Table 1:** Results of chemical elements mass fractions (mg/kg on dry mass basis) determined in *Mentha pulegium* L. by INAA and ICP-OES techniques.

Elements		*Mentha pulegium* L.	Techniques Used
1	Ba	26.39 ± 3.62	INAA
2	Br	67.14 ± 11.50	INAA
3	Ca	31,875.00 ± 47.00	ICP
4	Cl	2697.00 ± 5.00	INAA
5	Co	0.776 ± 0.060	INAA
6	Cr	10.11± 2.05	INAA; ICP
7	Cs	0.201 ± 0.038	INAA
8	Eu	0.054 ± 0.021	INAA
9	Fe	1603.90 ± 183.50	INAA; ICP
10	K	14,216.00 ± 4294.00	INAA
11	Mg	441.50 ± 5.00	ICP
12	Mn	71.200 ± 4.000	INAA
13	Mo	0.785 ± 0.256	INAA
14	Na	10,790.00 ± 544.00	INAA
15	Rb	5.83 ± 0.86	INAA
16	Sb	0.039 ± 0.013	INAA
17	Sc	0.022 ± 0.002	INAA
18	Sr	135.40 ± 9.33	INAA
19	Th	0.510 ± 0.147	INAA
20	U	0.063 ± 0.022	INAA
21	Zn	44.50 ± 2.00	INAA; ICP

Twenty-one elements (Ba, Br, Ca, Cl, Co, Cr, Cs, Eu, Fe, K, Mg, Mn, Mo, Na, Rb, Sb, Sc, Sr, Th, U and Zn) reported in our samples.

**Table 2:** Descriptive statistics of the mineral composition of *Mentha pugleum* L.

Specification	Ba	Br	Ca	Cl	Co	Cr	Cs	Eu	Fe	K	Mg	Mn	Mo	Na	Rb	Sb	Sc	Sr	Th	U	Zn
Mean	26.4	67.14	31,875	2697	0.78	10.1	0.20	0.05	1 603.9	14 216	441.5	71.20	0.79	10,790.00	5.83	0.04	0.02	135.40	0.51	0.06	44.50
Median	26.2	68.67	31,886	2695	0.76	9.09	0.22	0.05	1 649.6	12 943	441.7	70.40	0.83	10,655.00	5.75	0.04	0.02	134.00	0.48	0.07	43.80
SD	3.55	10.82	42.58	4.36	0.06	1.78	0.03	0.02	165.45	3 820	4.90	3.67	0.24	490.63	0.82	0.01	0.00	8.71	0.13	0.02	1.76
Skewness	0.18	−0.62	−1.08	1.63	1.44	1.73	−1.73	1.68	−1.15	1.33	−0.18	0.94	−0.84	1.14	0.43	1.07	−1.15	0.70	0.98	−0.85	1.51
Range	7.10	21.47	83	8.00	0.11	3.08	0.06	0.03	321.30	7315	9.80	7.20	0.47	953.00	1.64	0.02	0.00	17.26	0.26	0.04	3.30
V *	94.7	37.2	0.1	0.93	3221.6	247.	12,438	46,296.3	1.56	0.18	5.7	35.1	3184.	0.23	428.8	64,102.6	113,636.4	18.5	4901.0	39,682.5	56.2

* V—variability coefficient (%).

**Table 3:** The chemical composition of essential oil Mentha pulegium L. (aerial parts).

N°	Compounds	KI	HD		SD %		MAD %	
			TR *	HD %	TR	SD %	TR	MAD %
1	α-Thujene	931	0.000	0.000	0.000	0.000	13.014	0.01
2	α-Pinene	939	13.415	0.42	13.441	0.24	13.415	0.07
3	Cyclohexanone, 3-methyl	952	14.494	0.07	14.532	0.04	14.494	0.01
4	Camphene	953	0.000	0.00	0.00	0.00	14.397	0.01
5	Sabinene	976	16.28	0.06	0.000	0.00	16.275	0.01
6	β-Pinene	980	16.412	0.30	16.437	0.24	16.407	0.05
7	3-Octenone	986	17.408	0.20	17.439	0.17	17.418	0.04
8	Myrcene	991	17.731	0.16	17.757	0.11	17.726	0.01
9	3-Octanol	993	18.094	0.03	18.118	0.03	18.198	0.01
10	Pseudolimonene	1004	18.472	0.03	18.494	0.03	18.462	0.03
11	Limonene	1031	20.415	1.55	20.464	1.37	20.384	0.17
12	Terpinolene	1088	24.896	0.02	0.000	0.00	0.000	0.00
13	Linalool	1098	0.000	0.00	25.987	0.01	0.000	0.00
14	Menthone	1154	30.114	13.01	30.166	8.69	30.418	5.72
15	Isomenthone	1164	30.559	0.73	30.577	0.45	30.734	0.18
16	Cis-Isopulegone	1172	31.428	1.37	31.441	1.11	31.471	0.13
17	Menthol	1173	31.156	0.11	0.000	0.00	31.025	0.02
18	α-Terpineol	1189	33.356	0.14	34.004	0.18	32.965	0.02
19	Pulegone	1237	37.071	74.81	37.549	73.17	36.697	71.52
20	Menthyl acetate	1294	38.592	0.23	38.754	0.28	38.503	0.02
21	Piperitone	1340	42.972	3.82	43.247	5.77	42.869	0.39
22	(*E*)-Caryophyllene	1418	47.728	0.16	47.779	0.18	47.685	0.04
23	α- Humulene	1454	49.849	0.43	49.911	0.56	49.800	0.07
24	Germacrene D	1480	51.542	0.05	51.565	0.03	52.513	0.08
25	(-)-Caryophyllene oxide	1581	57.495	0.01	57.519	0.05	57.490	0.14
26	Humulene Oxide	1606	59.001	0.02	59.032	0.09	59.004	0.20
	Total Identified			97.73		92.80		78.95
	Yield of Essential Oil (*v*/*w*) %			0.204		0.200		0.175
	monoterpene hydrocarbons			2.54		1.99		0.36
	oxygenated monoterpenes			93.99		89.38		77.98
	sesquiterpene hydrocarbons			0.64		0.77		0.19
	oxygenated sesquiterpene			0.03		0.14		0.34
	other compounds			0.53		0.52		0.08

KI—experimentally determined; HD—hydrodistillation, SD—steam distillation; MAD—microwave distillation; * TR—the different compounds have been listed according to their retention times.

**Table 4:** Effect of the method of extracting plant material on the yield and composition of essential oils of the aerial parts of *Mentha pulegium* in comparison with the results of other authors.

Compounds	Ingredients of Essential Oils (%)					Yield of Oil (*v/w*) or (*w/w*)		Distillation Technique	Plant Organs	Form
	Limonene	Menthone	Menthol	Pulegone	Piperitone					
Distillation technique	1.55 ^a,^*	13.01 ^a^	0.11 ^a^	74.81 ^a^	3.82 ^b^	0.204 ^a^	*v/w*	HD %	Aerial parts	fresh
	1.37 ^a^	8.69 ^b^	0.00 ^a^	73.17 ^a^	5.77 ^a^	0.200 ^a^	*v*/*w*	SD %	Aerial parts	fresh
	0.17 ^b^	5.72 ^c^	0.02 ^a^	71.52 ^b^	0.39 ^c^	0.175 ^b^	*v*/*w*	MAD %	Aerial parts	fresh
Mean	1.03	9.44	0.04	73.17	3.33	0.190	-	-	-	-

* The presence of the same letter index by the means (at least one) means that there is no statistically significant difference between them. The subsequent letter indices a, b, c, define the groups in ascending order.

**Table 5:** Total phenolics and flavonoids of *Mentha pulegium* extraction rate in methanol extract.

Specification	Symbol	T1	T2	T3	Mean	LSD_0.05_
Phenolic compounds (mg EAG/g extrait) (TPC) *	TPC	22.79 b ***	25.15 a	25.53 a	24.49 ± 1.48	1.41
Flavonoid content (g QE/g extrait) TFC **	TFC	19.35 a	17.97 b	18.99 a	18.77 ± 0.72	0.94
Rate of methanol extraction (%)	R%	15.61 a	14.57 b	15.33 b	15.17 ± 0.54	0.76

Harvesting dates for aerial parts: T1—before flowering, T2—at the beginning of flowering and T3—at full flowering *Mentha pulegium*. TPC *—phenolic compounds (mg EAG/g extrait); TFC **—flavonoid content (g QE/g extrait). *** The presence of the same letter index by the means (at least one) means that there is no statistically significant difference between them. The subsequent letter indices a, b, c, define the groups in ascending order.

**Table 6:** Descriptive statistic of total phenolics and flavonoids of *Mentha pulegium* extraction rate in methanol extract.

Specification	TPC *	TFC **	R *** (%)
Mean	24.49	18.77	15.17
Median	25.15	18.99	15.33
Standard deviation	1.48	0.72	0.54
Skewness	−1.61	−1.25	−1.22
Range	2.74	1.38	1.04
Minimum	22.79	17.97	14.57
Maximum	25.53	19.35	15.61
Coefficient of variation V (%)	6.06	3.81	3.55

TPC *—phenolic compounds (mg EAG/g extract); TFC **—flavonoid content (g QE/g extract) and R ***: Rate of methanol extraction.

**Table 7:** Antioxidant power of essential oils and methanolic extracts of the plants studied, in vitro, via the inhibition of DPPH.

Specification	Symbols	DPPH IC_50_ (μg/mL)	ABTS IC_50_ (μg/mL)
Essential Oil	MP oil	25,682.7	7428.5
Methanolic extract	MP	238.48	nd *
Compose of Reference	V.E (α-tocopherol)	15.81	37.79
	Butylhydroxyanisol (BHA)	15.21	nd

* nd (not determined).

**Table 8:** Antibacterial activity of essential oils and methanolic extracts of *Mentha pulegium* L., against bacteria and yeast strains, in vitro.

Specification	Concentration (µL/disc)		Our Data Diam (Diameter Inhibition) (mm), MIC (Minimal Concentration Values) (µL/mL) and MBC (Minimal Bactericidal Concentration) (µL/mL)					Literature		
			*Mentha pulegium* L.			References Used		[37]	[38]	[39]
	Groups		MPHD	MPSD	MPex			MPHD	MPHD	MPHD
Gram positive	*Staphylococcus aureus* ATCC 6538	**Diam** Mean ± SD (mm)	37.33 ± 1.15	24.33 ± 0.58	14.33 ± 0.29	**Fosfomycin** 44 ± 0.5	**Carbenicilli** 37.5 ± 0.4	21	21.4 ± 0.8	23
		**MIC µL/ml**	1	2.5	nd *	nd	nd	0.5	nd	1.25
		**MBC µL/ml**	2	5	nd	nd	nd	0.5	nd	2.5
	*Bacillus subtilis* ATCC 6633	Diam Mean ± SD (mm)	25.5 ± 1.32	26.33 ± 1.53	15.66 ± 0.58	**Erythromycin** 32.5 ± 0.3	**Cephalexin** 31 ± 0.3	nd	nd	24
		**MIC µL/ml**	20	20	nd	nd	nd	nd	<30	2.5
		**MBC µL/ml**	20	20	nd	nd	nd	nd	1	2.5
Gram negative	*Pseudomonas aeruginosa* ATCC 9027	Mean ± SD (mm)	16.67 ± 0.58	13.67 ± 0.57	10.17 ± 0.29	**Fosfomycin** 31 ± 0.21	Fosfomycin 33.2 ± 0.31	nd	0	<8
	*Escherichia coli* ATCC 8739	Diam Mean ± SD (mm)	31.67 ± 1.53	25.67 ± 0.58	14.67 ± 0.58	Fosfomycin 44 ± 0.31	Fosfomycin 37.5 ± 0.31	nd	12.6 ± 0.5	28
		**MIC µL/ml**	5	20	nd	nd	nd	4	1	1.25
		**MBC µL/ml**	5	20	nd	nd	nd	4	10	1.25
**Yeast**	*Candida albicans* ATCC 10231	Mean ± SD (mm)	11 ± 0.87	9 ± 0	16 ± 0.87	nd	nd	16	nd	19

*MPHD* (EO extracted by hydrodistillation), *MPSD* (EO extracted by steam distillation), *MP* (methanol extraction), Diam (Diameter inhibition (mm)), MIC (Minimal concentration values (µL/mL)) and MBC (Minimal bactericidal concentration (µL/mL)), * nd (not determined).

**Table 9:** Result of anti-inflammatory activity *in vivo*.

Specification	Average Paw Weight (g)		% Edema	% Edema Reduction
	Left	Right		
Witness “Physiological water”	0.109 ± 0.006	0.076 ± 0.004	43.42%	0%
Reference “Diclofenac sodium”	0.171 ± 0.009	0.142 ± 0.001	20.42%	52.97%
MP aqueous extract	0.190 ± 0.001	0.142 ± 0.001	33.80%	22.16%

**Table 10:** Effect of the method of extracting plant material on the yield and composition of essential oils of the aerial parts of Menth’s pulegium in comparison with the results of other authors.

Compounds	Ingredients of Essential Oils (%)					Yield of Oil (*v*/*w*) or (*w*/*w*)		Distillation Technique	Plant Organs	Dried or Fresh Form
	Limonene	Menthone	Menthol	Pulegone	Piperitone					
Algeria [44]	2.10	0.40	nd	76.90	1.30	0.910	*v*/*w*	HD	Aerial parts	dried
Algeria [45]	4.29	19.24	nd	38.82	6.35	1.000	*v*/*w*	HD	Aerial parts	dried
Morocco [38]	0.10	0.10	nd	69.80	3.10	2.700	*v*/*w*	SD	Aerial parts	dried
Morocco [46]	0.20	4.80	nd	74.60	nd	2.000	*w*/*w*	HD	Aerial parts	dried
Egypt [30]	1.31	nd	nd	43.46	12.20	1.500	*v*/*w*	SD	Leaves	dried
Portuga l [47]	0.40	9.00	1.3	80.60	0.04	nd	*v*/*w*	HD	nd	nd
Italy [48]	0.70	1.00	nd	86.20	nd	nd	*v*/*w*	HD	Aerial parts	nd
Croatia [49]	1.20	14.00	nd	54.40	3.7	nd	*v*/*w*	HD	Aerial parts	fresh
Iran [50]	0.00–3.60	0.20–29.60	0.00–4.4	3.70–51.7	0.00–4.90	0.220–1.630	*v*/*w*	HD	Aerial parts	dried
Chile [29]	nd	20.48	28.79	29.33	nd	1.30	*v*/*w*	HD	Aerial parts	fresh
Turkey [51]	0.3	40.2	0.20	27.7	12.5	1.60	*v*/*w*	SD	Aerial parts	nd
Bulgaria [52]	0.00–1.20	1.1–5.8	nd	27.2–49.7	5.1–6.5	0.01–1.54	*v*/*w*	SD	Aerial parts	dried

**Table 11:** Comparison of our result with studies of total phenolic content (TPC *), total flavonoid content (TFC **) and the yield of methanolic extracts (R *** (%)) for the aerial parts of *Mentha pulegium* L.

Plant	Current Study		Previous Studies			
*Mentha* *pulegium* L.			El-Tarf [35]	Tunisia [43]	Croatia [15]	Turkey [56]
	TPC *	24.49	17.00	37.4	157.92	97.2
	TFC **	18.77	13.72	33.83	18.58	20.88
	R *** (%)	15.17	Nd	24	10.68	25.85

Nd: not analyzed.

## Data Availability

All data generated or analyzed are contained within the present article.
